# Analysis of depressive episodes, their recurrence and pharmacologic treatment in primary care patients: A retrospective descriptive study

**DOI:** 10.1371/journal.pone.0233454

**Published:** 2020-05-21

**Authors:** Shysset Nuggerud-Galeas, Loreto Sáez-Benito Suescun, Nuria Berenguer Torrijo, Ana Sáez-Benito Suescun, Alejandra Aguilar-Latorre, Rosa Magallón Botaya, Bárbara Oliván Blázquez

**Affiliations:** 1 Institute for Health Research Aragón, Zaragoza, Spain; 2 University San Jorge, Zaragoza, Spain; 3 Department of Medicine, Psychiatry and Dermatology, University of Zaragoza, Zaragoza, Spain; 4 Department of Psychology and Sociology, University of Zaragoza, Zaragoza, Spain; University of Oslo, NORWAY

## Abstract

**Background:**

Depression is one of the most prevalent health problems, frequently being a medium- and long-term condition, with a high comorbidity rate and with frequent relapses and recurrences. Although numerous studies have compared the effectiveness of specific antidepressant therapy drugs and have assessed relapses, scientific evidence on the relationship between pharmacologic treatments and recurrence is scarce. The objective of this study is to describe depressive episodes in a primary care patient cohort, the percentage of depression recurrences and the administered pharmacologic treatment, from a naturalistic perspective.

**Methods:**

Retrospective descriptive study. 957 subjects were included. The dependent variable was a depression diagnosis and independent variables were: gender, age at time of data collection; age of onset, first-episode treatment, number of recurrences, age at recurrences, treatment prescribed for recurrences using therapeutic groups categorization.

**Results:**

Recurrences are frequent, affecting more than 40% of the population. In the first episode, 13.69% of the patients were not prescribed pharmacological treatment, but this percentage decreased over the following depression episodes. 80.9% of the patients who did not receive drug treatment in the first depression episode did not experience subsequent episodes. Monotherapy, and specifically, SSRIs were the most frequently prescribed treatment option for all depressive episodes. Regards the combined pharmacologic treatment, the most frequent drug combinations were SSRIs and benzodiazepines.

**Limitations:**

In order to increase the power of results, the statistical analysis was performed using therapeutic groups categorization, not individually analyzing each drug and dose.

**Conclusions:**

Depressive episode recurrence is frequent in primary care patients. Further studies having a prospective design are needed in order to expand on this issue.

## Introduction

Depression is one of the most prevalent health problems in primary care, [1–3] and is one of the four medical conditions causing the largest number of disabilities [1,4,5]. Women typically have a two-fold increased risk of major depression as compared to men [6], and the peak age of risk for the first onset of a major depressive episode (including bipolar depression) is estimated to range from mid-late adolescence to the early 40s [7]. It is also the most expensive mental disorder, accounting for 1% of the Gross Domestic Product (GDP) of the European Union [8–10]. This mental health problem is often a medium- and long-term condition, having a high comorbidity rate [11,12].

Major depressive disorder (MDD) is highly recurrent [2,3,13], making the prevention of depressive relapse and recurrence depression one of the most important challenges to its management [14]. The ‘MacArthur Research Network on the Psychobiology of Depression’ proposed operational criteria for the constructs of response, remission, recovery, relapse and recurrence [3,15,16] as guidance for the field. According to these criteria, remission occurs when symptoms have largely normalized and the patient can be considered well for a given period of time (often defined as two months or longer), and precedes both recovery and recurrence. Relapse suggests the reemergence of depression symptoms (presumably part of the depressive episode) following some degree of remission but preceding recovery; and recurrence implies the onset of a new depression episode following an extended period of remission, sufficiently long so as to assume that recovery had taken place.

Recurrences occur frequently [17,18]. Individuals suffering from an initial depressive episode have a 40% to 60% chance of experiencing a subsequent episode; individuals with 2 episodes have approximately 60% to 70% possibility; and for individuals experiencing three episodes, the risk is up to 90% [3,17,19,20]. The mean time of the recurrence following the initial episode is approximately three years, and subsequent episodes usually appear after 1 or 1.5 years. Recurrence risk is higher during the initial months following recovery [19]. Subsequently, mean time of recurrence progressively decreases, and length of recovery increases; in a study [21] performed on a Dutch population, patients suffering from major depression who recovered presented a cumulative recurrence range of 13% in 5 years, 23% in 10 years and 42% in 20 years, respectively.

Previous bibliographies have highlighted certain factors involved in the recurrence of depression, such as the severity of major depression episodes (MDE) [22,23]; the number of depressive episodes experienced [22–24], early age of onset of first MDE [22], comorbidity with axis I and axis II psychiatric pathology [22]; change in the size of specific brain areas [25]; immunologic irregularities [26]; history of suicide attempts [22]; family history of depression [27]; concurrent physical health problems and psychosocial difficulties [28]; stressful live events [29], and female gender and older age [22].

As for drug therapy, interruption of antidepressant treatment when in remission from the depressive episode leads to a risk of recurrence in 40–50% of the patients, whereas maintaining the treatment for 6 to 12 months after remission reduces this risk to 13–20% [30–32].

Although many studies have compared the effectiveness of specific antidepressant therapy drugs and have assessed relapses, there is little scientific evidence regarding the relationship between pharmacologic treatments and recurrence. To our knowledge, no studies have analyzed the relationship between antidepressant pharmacologic strategies and depression recurrence. Only a few meta-analyses include studies of specific drugs versus placebo, obtaining inconclusive conclusions [33–35]. Therefore, there is insufficient knowledge on the association between the different classes and combinations of psychoactive drugs and the occurrence of such relapses and recurrences.

Primary health care services are the ideal setting for carrying out recurrence prevention strategies. More than 80% of all patients suffering from depression are currently managed by primary health care and drug therapy is the first-line treatment. So, antidepressant consumption has increased over recent years in Europe [36,37]. Antidepressant use has been reported to be higher in women and it tends to increase with age [38], with selective serotonin reuptake inhibitors (SSRIs) being the top choice, according to clinical practice guidelines [39].

Therefore, the aim of this study is to describe depressive episodes in a primary care patient cohort, the percentage of depression recurrences and the administered pharmacologic treatment, from a naturalistic perspective. This article provides comprehensive insights into antidepressant trends and patterns over a 16-year period, considering the recurrence of depressive episodes.

## Methods

### Design

Retrospective observational cohort study.

### Study population and sample size

Data were obtained from the clinical histories of patients over the age of 18 at a primary care center in Zaragoza (Spain) between October 2017 and February 2018. This health care center attends to 18,875 patients. From this population, patients were selected based on the following inclusion criteria: 1) Aged 18 and over; 2) having been diagnosed with major depression (codified as depressive disorder according to the World Organization of Family Doctors (WONCA) ICPC-2) diagnosed by his/her primary care physician between 2001 and 2017. Exclusion criteria were: 1) Database inconsistencies; 2) having a clinical history of less than two years at the time of the study; 3) having a severe psychiatric disorder comorbidity (schizophrenia or bipolar disorder); 4) presenting dysthymia and 5) patients referred to and followed up on by the psychiatric unit.

The population cared for in this health center is aged, having an aging rate of 224.65%, an over-aging rate of 17.53%, and with 22.05% being immigrants. The percentage of males is 47.60%, having a mean age of 45 years and 10 months, whereas women account for 52.40%, with a mean age of 48 years and 10 months. The percentage of the population aged 25 and younger is 21.25% and those over the age of 60 account for 27.2% [40].

**Fig 1:**
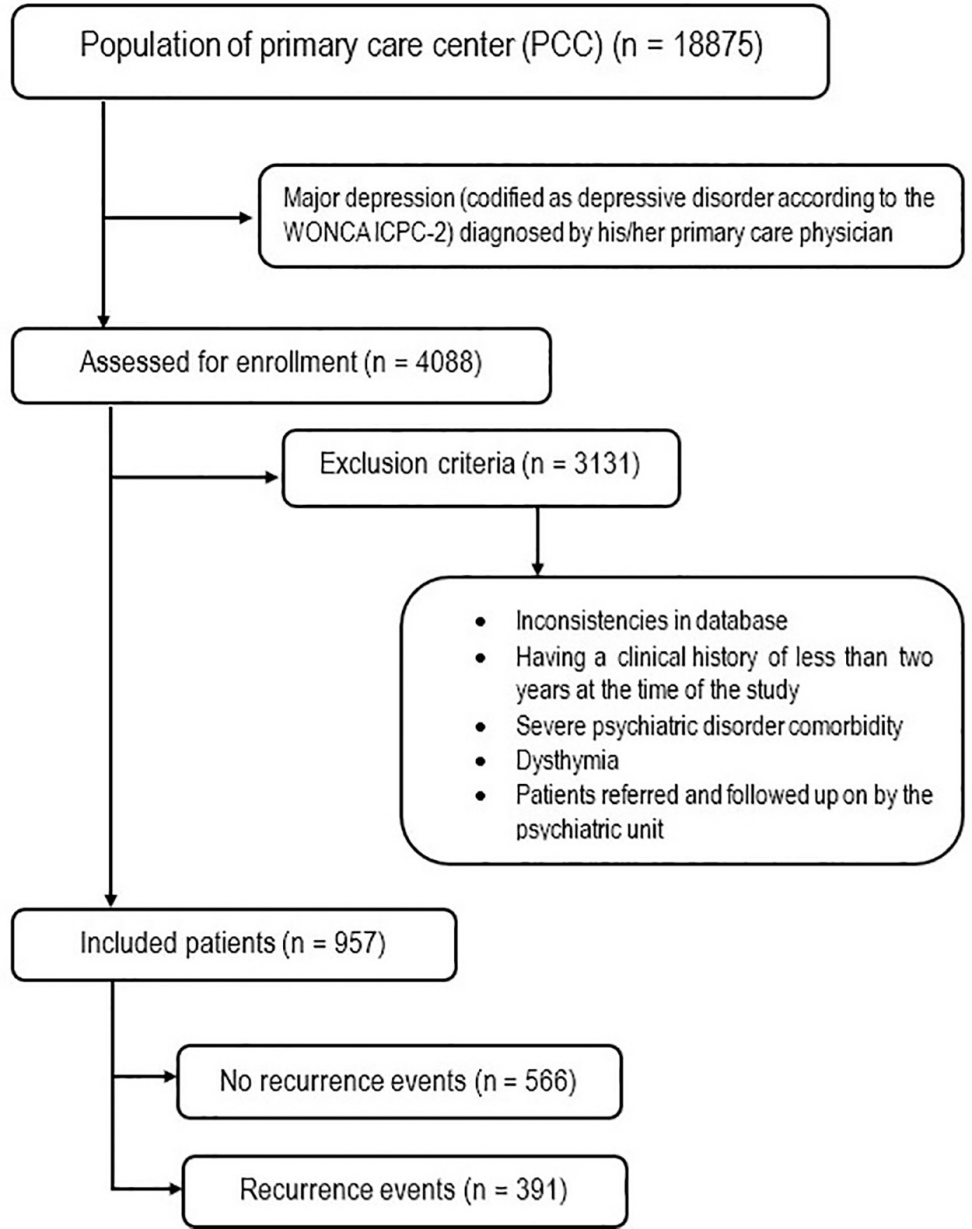
Flowchart of the sample.

The sample size included 957 subjects meeting the selection criteria. Fig 1 shows the flowchart of the sample size.

### Study variables and data collection

Data were collected retrospectively from the computerized clinical histories of all primary care patients assigned to the selected health care center. The dependent variable was the depression diagnosis (The P76 International Classification of Primary Care 2° edition (ICPC-2) code of the World Organization of Family Doctors (WONCA) was used). The definition of recurrence created by Frank et al. and followed in various studies [3,14,16,17] has been used. Therefore, recurrence is considered to be a diagnosis made by the family physician, for a new depression episode (after having suffered an initial episode) in the case in which there was a previous remission of a sufficient duration so as to assume that recovery had occurred. A minimum period of 6 months following the end of treatment, according to the computer-based clinical history, was considered for the definition of a recurrence episode. Since this is a retrospective study, no validation has been conducted for the diagnosis made by the family physician.

Independent variables were gender, age at time of data collection, age of onset, first-episode treatment prescription and Anatomical Therapeutic Chemical (ATC) drug code, recurrence and number of recurrences, age at recurrences, treatment prescribed for recurrences and drug ATC code, and time to recurrences. Pharmacologic treatment has been grouped according to therapeutic group (ATC classification) [41] and is shown in Table 1. Benzodiazepines were collected, despite not being antidepressants, since they are frequently prescribed for this psychopathology. Using the patient’s clinical history, it is possible to determine if the prescription is collected from the community pharmacies, so the family physician can assess the therapeutic adherence and intervene when considered necessary. In cases in which the family physician did not prescribe any pharmacological treatment, based on his/her clinical criteria, this has also been noted.

**Table 1:** Pharmacologic treatment according to therapeutic group (ATC classification).

Therapeutic group	Active ingredient
Benzodiazepines	Alprazolam/Trankimazin, Bentazepam, Bromazepam, Clonazepam, Clorazepate Dipotassium, Diazepam, Midazolam, Ketazolam, Lorazepam, Lormetazepam, Medazepam, Piracetam, Tepazepam, Halazepam/Flurazepam
Selective Serotonin Reuptake Inhibitors (SSRIs)	Citalopram, Escitalopram, Fluoxetine, Paroxetine, Sertraline, Fluvoxamine
Tricyclic Antidepressants (TCAs)	Clomipramine, Imipramine, Amitriptyline, Tianeptine, Doxepine, Nortriptyline
Serotonin–norepinephrine reuptake inhibitors (SNRIs)	Desvenlafaxine, Duloxetine, Venlafaxine
Norepinephrine reuptake inhibitors (NRIs)	Reboxetine
Heterocycles	Mianserin, Maprotiline
Noreprinephrine-dopamine reuptake inhibitors (NDRIs)	Bupropion
Serotonin antagonist and reuptake inhibitors (SARIs)	Trazodone
Noradrenergic and specific serotonergic antidepressants (NaSSAs)	Mirtazapine
Melatonin receptor agonist (MRA)	Agomelatine
Serotonin activity modulator (SMS)	Vortioxetine
Hypnotics	Zolpidem
Antiepileptic	Topiramate
Typical Neuroleptics	Levomepromazine, Haloperidol, Trifluoperazine
Atypical Neuroleptics	Quetiapine, Risperidone, Olanzapine
Non-specific drugs	Melitracen/flupentixol, Deanol/heptaminol, Vitamin B complex, Piridoxine/Sulbutiamine, Levosulpiride/sulpiride, Gabapentin

These variables were collected retrospectively from 2018 to 2001. The cut-off point was 2001, since this was the year as of which the computer-based clinical history was available and fully established at this health center. Given the universal nature of the health system and the absence of other primary health care providers, the data obtained in the study is considered to be representative of almost 100% of the population that met the study’s inclusion criteria.

### Statistical analysis

A descriptive analysis of the sample was conducted using means and standard deviations for continuous data and frequencies and percentages for categorical data. The following non-parametric tests (Shapiro-Wilk test p-value < 0.001) were used to analyze the relationship between demographic variables and recurrence apparition (yes/no) and the number of recurrences: Mann–Whitney U and Kruskal Wallis for numerical variables, and Chi-square and Fisher tests for categorical ones. Pharmacologic treatments were grouped into ATC categories to provide a more robust analysis.

### Ethical considerations

The authors declare that all procedures contributing to this work comply with the ethical standards of the Declaration of Helsinki of 1975, as revised in 2008. The study protocol was approved by the Clinical Research Ethics Committee of Aragón (Spain) (PI18/029) and by the Aragón Health Service. The database of the pharmacological treatment was facilitated anonymously by the Aragon Healthcare Department. This study forms a part of another study whose main objective is to analyze the risk factors for the recurrence of a major depression episode included in the literature and specifying the weight of each of these in a sample of subjects that have experienced a major depression episode during their life. Patients wishing to participate were requested to sign an informed consent form for data collection via questionnaire.

## Results

A total of 957 subjects were diagnosed with major depression during the study period. Of these, 708 (73.98%) were women and 249 (26.02%) were men. Their mean age was 60.95 years (SD: 17.33). As for the number of recurrences, more than half, 59.1% (566 patients) did not have recurrence events, while 40.9% (391 patients) experienced a second depressive episode. A third depression diagnosis took place in 14.8% (142 patients) and 3.8% (36 subjects) experienced a fourth depression event. Therefore, of the patients having a second episode of depression, 36.31% experienced a third event, and of these, 25.32% had a fourth depression diagnosis. The p-value (*tendency*) revealed an increase in age as the number of recurrences increased (p(*tendency*) < 0.001). The mean time between the first and second episodes was 4.51 years (SD: 3.35), 3.75 years (SD: 2.23) between the second and third episodes, and 3.53 years (SD: 2.23) between the third and fourth episodes. Significant differences were found between men and women, both in number of recurrences and percentage of subjects suffering from depression recurrences. The number of recurrences and age of first episode are higher for women. All of these differences are shown in Table 2.

**Table 2:** Differences between men and women in number of recurrences; mean age at diagnosis of depressive episodes and time to recurrences.

	N (men / women)	MEN% / Mean (SD)	WOMEN% / Mean (SD)	p-value
**Number of recurrences**	249/708	0.48 (0.73)	0,63 (0.85)	0.022
**1**^**st**^ **recurrence (%)**	88/303	35.34%	42.79%	0.040
**2**^**nd**^ **recurrence (%)**	28/114	11.24%	16.10%	0.064
**3**^**rd**^ **recurrence (%)**	4/32	1.60%	4.51%	0.038
**Age 1**^**st**^ **at diagnosis**	249/708	49.89 (17.62)	52.95 (17.10)	0.017
**Age 2**^**nd**^ **at diagnosis**	88/303	55.78 (18.33)	58.42 (16.35)	0.280
**Time between 1**^**st**^ **and 2**^**nd**^ **diagnosis**	88/303	4.97 (3.78)	4.37 (3.21)	0.268
**Age at 3**^**rd**^ **diagnosis**	28/114	62.64 (17.83)	61.61 (15.47)	0.667
**Time between 2nd and 3**^**rd**^ **diagnosis**	28/114	3.57 (2.55)	3.79 (2.59)	0.589
**Age at 4**^**th**^ **diagnosis**	4/32	59.25 (20.10)	63.97 (13.48)	0.752
**Time between 3**^**rd**^ **and 4**^**th**^ **diagnosis**	4/32	3.75 (3.20)	3.50 (2.15)	0.942

Statistic used: Mann–Whitney U (Chi square and Fisher test were used for the percentage of subjects suffering a first, second or third recurrence).

In the first episode, 13.69% of the patients were not prescribed pharmacological treatment, based on the criteria of their family physician; 45.45% were prescribed drugs for depression treatment in monotherapy, and 40.86% were prescribed polytherapy drug treatment. As for the patients who were not prescribed drug treatment in the first depression episode, their percentage rose to 13.69%, but it decreased over the following depression episodes. 80.9% of the patients who did not receive drug treatment in the first depression episode did not experience subsequent episodes; 16% experienced one subsequent episode and 3.1% experienced two subsequent episodes.

As for pharmacologic treatment, SSRIs and benzodiazepines, specifically paroxetine, escitalopram and lorazepam were the most frequently prescribed drugs for all depression episodes. This latter drug was prescribed mainly in combination with other medications. Paroxetine was prescribed to 27.48% of the patients in their first episode, to 26.11% in their second, to 25.92% in their third and 26.66% in their fourth episode. Escitalopram was prescribed to 14.048% of the patients in the first episode, to 18.61% in the second, to 26.67% in the third and to 26.66% in the fourth episode.

As for anxiolytics, the second therapeutic group prescribed as a complementary antidepressant treatment, lorazepam was prescribed to 26.27% of the patients in the first episode, to 21.66% in the second, to 21.48% in the third and to 23.33% in the fourth episode.

Monotherapy, and specifically, SSRIs were the most frequently prescribed treatment option for all depressive episodes. In all depression episodes, patients were mainly treated with a SSRI. Benzodiazepines and SNRIs were also prescribed slightly more often than the other therapeutic groups. No prescriptions of NRI, MRA or typical neuroleptics were prescribed in monotherapy.

As for combined pharmacologic treatment, in the first depression episode, a total of 50 different drug combinations were found: 29 combinations of drugs and 21 combinations of three medicines. There were 13 drug combinations prescribed to 86.96% of the patients via polytherapy. In the other depression episodes, the number of combinations was lower. The most frequent drug combinations were SSRIs and benzodiazepines. Tables 3–5 describe all types of treatments, both in monotherapy and polytherapy, for each depression episode.

**Table 3:** Description (frequency and percentage) of treatment types (non-pharmacologic, monotherapy and polytherapy) for each depressive episode.

Treatment	1^st^ episode Frequency (%) N = 957	2^nd^ episode Frequency (%) N = 391	3^rd^ episode Frequency (%) N = 142	4^th^ episode Frequency (%) N = 36
**Non-pharmacologic**	131 (13.69%)	31 (7.92%)	7 (4.93%)	6 (16.66%)
**Monotherapy**	435 (45.45%)	206 (52.69%)	83 (58.45%)	19 (52.78%)
**Polytherapy**	391 (40.86%)	154 (39.39%)	52 (36.62%)	11 (30.56%)

**Table 4:** Description (frequency and percentage) of pharmacologic treatment (ATC category) in monotherapy for each depression episode.

Pharmacologic treatment	1^st^ episode Frequency (%) N = 435	2^nd^ episode Frequency (%) N = 206	3^rd^ episode Frequency (%) N = 83	4^th^ episode Frequency (%) N = 19
**Benzodiazepine**	47 (10.76%)	18 (8.74%)	6 (7.23%)	2 (10.53%)
**SSRI**	290 (66.66%)	138 (66.99%)	55 (66.27%)	11 (57.89%)
**TCA**	9 (2.06%)	2 (0.97%)	1 (1.20%)	1 (5.26%)
**SNRI**	40 (9.15%)	21 (10.19%)	15 (18.07%)	2 (10.53%)
**Heterocycle**	5 (1.14%)	1 (0.49%)		
**NDRIs**	3 (0.69%)	1 (0.49%)		
**SARI**	2 (0.46%)	4 (1.94%)	4 (4.82%)	1 (5.26%)
**NaSSA**	13 (2.97%)	9 (4.37%)		1 (5.26%)
**SMS**	1 (0.23%)	1 (0.49%)		1 (5.26%)
**Hypnotic**	3 (0.69%)	5 (2.43%)		
**Anti-epileptic**		1 (0.49%)	1 (1.20%)	
**Atypical neuroleptics**	3 (0.69%)	1 (0.49%)		
**Non-specific medicines**	19 (4.35%)	4 (1.94%)	1 (1.20%)	

SSRI, Selective Serotonin Reuptake Inhibitors; TCA, Tricyclic Antidepressants; SNRI, Serotonin–norepinephrine reuptake inhibitors; NDRI, Noreprinephrine-dopamine reuptake inhibitors; SARI, Serotonin antagonist and reuptake inhibitors; NaSSA, Noradrenergic and specific serotonergic antidepressants; SMS, Serotonin activity modulator.

**Table 5:** Description (frequency and percentage) of combined pharmacologic treatment (ATC classification) for each depressive episode.

Pharmacological polytherapy	1^st^ episode Frequency (%) N = 391	2^nd^ episode Frequency (%) N = 154	3^rd^ episode Frequency (%) N = 52	4^th^ episode Frequency (%) N = 11
**Combination of 2 drugs**				
SSRI+ benzodiazepine	218 (55.76%)	79 (51.3%)	28 (53.85%)	8 (72.73%)
SSRI+ non-specific medicine	16 (4.10%)			
SSRI+ NaSSA	6 (1.53%)	4 (2.60%)	4 (7.69%)	
SSRI+ hypnotic	15 (3.83%)			
TCA + Benzodiazepine	8 (2.05%)			
SNRI + Benzodiazepine	32 (8.18%)	14 (9.09%)	4 (7.69%)	
SNRI+NaSSA		5 (1.95%)		
Heterocycle + Benzodiazepine	4 (1.02%)			
Nonspecific medicine + Benzodiazepine	8 (2.05%)			
NaSSA + Benzodiazepine	5 (1.28%)			
SARI + Benzodiazepine		4 (2.60%)		
Benzodiazepine + Benzodiazepine	7 (1.79%)	4 (2.60%)		
**Combination of 3 drugs**				
SSRI + 2 Benzodiazepine	12 (3.07%)			
SSRI + non-specific medicines + Benzodiazepine	5 (1.28%)			
SNRI + NaSSA + Benzodiazepine	4 (1.02%)			
SSRI + hypnotic + Benzodiazepine		4 (2.60%)		
Other combinations with a frequency < = 3 subjects	51 (13.04%)		16 (30.77%)	3 (27.27%)

SSRI, Selective Serotonin Reuptake Inhibitors; TCA, Tricyclic Antidepressants; SNRI, Serotonin–norepinephrine reuptake inhibitors; SARI, Serotonin antagonist and reuptake inhibitors; NaSSA, Noradrenergic and specific serotonergic antidepressants.

## Discussion

The results of this retrospective cohort study reveal a percentage of the population in which recurrence of depressive episodes occurs frequently. In our study, 40% of all patients suffered at least one recurrence. The study by Solomon et al. [20] revealed higher numbers; 66% of the subjects having an initial depression episode experienced at least one recurrence during 10 years of follow up. Other studies [16,19,42] however, have suggested a lower prevalence, with recurrences taking place in approximately 33%, a figure which is more in line with our study.

As for the age in which the depression episodes were diagnosed, we have found high figures, given that the mean age at first diagnosis for men is 49.89 (SD: 17.62) and 52.95 in women (SD: 17.10). In the study by Kessler and Bromet [6], the WHO World Mental Health (WMH) survey revealed that the median retrospectively-reported age of onset of depression episodes was the mid-20s, with the interquartile range indicating that across all countries, the peak risk period for the onset of MDD ranged from mid-late adolescence to the early 40s. In a study by Bockting et al [3], based on the World Mental Health Survey Initiative Version of the World Health Organization Composite International Diagnostic Interview (WMH-CIDI), the median age of onset (i.e., 50th percentile of the age-of-onset distribution) was the 30s for mood disorders. However, of the high income countries, Spain has one of the latest median ages of onset, at 30.0 years [6]. However, there may be other causes for this, including the failure of family physicians to diagnose younger patients, given the high population of immigrants in the healthcare system who are older [40], or since there may have been prior episodes that were not considered and that may have been omitted when transferring the clinical histories to electronic means due to patient mobility between health centers. As for the failure to diagnose, under-diagnosis and a difficulty of diagnosis occur in primary healthcare centers [43,44], since it is based on criteria and often not on very specific symptoms, which may be difficult to recognize by poorly trained professionals [45,46]. Although the most common stage of onset for mood disorders occurs during early adulthood (specifically, at the age of 30) [2], young adults do not tend to seek out health assistance given the related social stigma [47] or, in the case of young men from deprived backgrounds, they often fail to recognize a mental health problem and are less likely to suggest visiting a doctor than women [48].

In our research, the mean time between the first and second episode was 4.51 years (SD: 3.35), and this period was lower as the number of recurrences increased. This tendency has been seen previously in the bibliography, although the mean time of recurrence was lower, approximately 3 years for the time between the first and second episode and 1 to 1.5 years for subsequent episodes (Solomon et al., 2000). Additional research on the role of age as a sociodemographic factor is necessary, since in our study, a positive relationship was found between the number of recurrences and age, while in a review of naturalistic cohort studies [16], no association was found between this factor and the possibility of recurrence.

As for patients who are not prescribed drug treatment, this decision, made by the family physician, to not prescribe anything, may be related to the severity of the depressive episode, since disorder severity has been correlated with the probability of treatment [49]. In addition, clinical practice guidelines [32,50] have established recommendations for gradual treatment, based on scientific evidence; they recommend that the onset of intervention be the less intrusive of those that have been proven effective. In the computerized clinical history, the severity of the depression episode is not included. Therefore, it has been impossible to analyze whether these patients who did not receive pharmacological treatment were those having the mildest symptomology. A high percentage of these patients, (over 80%), however, did not experience recurrence.

According to current recommendations for antidepressants in patients suffering from unipolar depression [39], SSRIs and SNRI, more recently Duloxetine, Venlafaxine, and Desvenlafaxine, are the first line of treatment. These antidepressants were the most frequently consumed in our sample of patients, so we can assume that treatment for depression in primary care is consistent with clinical practice guideline recommendations.

Despite scientific evidence on the effectiveness and safety of these treatments, few studies have analyzed the relationship between antidepressant treatment and the recurrence of depression. In a meta-analysis conducted to establish the effectiveness of antidepressant treatment in preventing recurrence, 37 studies were analyzed, with 7,253 patients and comparing 24 different antidepressants with placebo. In 35 of these studies, antidepressant treatment was more effective than the placebo at preventing recurrence (RR = 2.03, CI 1.80–2.28, NNT = 3.8; p < 0.0001), but no significant differences were observed regarding the different treatments analyzed [33]. Nevertheless, other research has found variations based on the administered treatment. In a double-blind study analyzing time to recurrence appearance, fluoxetine was found to produce a longer time to recurrence as compared to venlafaxine [51]. In another study in which the effectiveness of treatment was analyzed, in terms of 24 months without recurrences [52], it was found that bupropion and fluvoxamine were the least effective in preventing recurrence of depression. In this study, and in line with our results, duloxetine was more effective in this sense. The authors conclude that the balance between dopamine, serotonin and noradrenaline may play a major role in the prevention of recurrences. Thus, an increased dopaminergic activity may not be useful in this sense, as occurs with the use of bupropion alone. However, a dual action, such as treatment with SNRI, could provide protection from recurrences.

Based on this theory, in our cohort of patients, SNRI and NaSSA were the only treatments that were not related to recurrences. Both therapeutic groups present a different mechanism of action, but they share a simultaneous and specific action over serotonin and noradrenaline. Future studies should attempt to demonstrate this hypothesis.

With respect to the prescription of benzodiazepines, insomnia has been described as a potential modifier of antidepressant treatment response [53] and anxiety disorder has been found to have a direct negative effect on the overall course of the disease and the lack of response to pharmacological treatment [54–56]. A randomized double-blind study revealed that high levels of anxiety not only increase failure of acute treatments but also raise the risk of recurrences over the two years following episode remission, despite being used as an optimal antidepressant therapy [54]. This suggests that the preventive effect of SNRIs and NaSSAs can be neutralized depending on the nature of the depressive episode.

This study has numerous strengths, but it also faces certain limitations. On the one hand, it examines recurrences in a cohort of patients across a 16-year-period, not only from an epidemiologic point of view, but also based on pharmacologic treatment, providing a more comprehensive view of this phenomenon. Its limitations mainly relate to the information source, since some relevant data were not registered in the database, such as length of the treatment and duration of the depressive episode, or the severity of the same, and were not considered in the research. Severity of the depressive episode is not usually collected by primary care physician. The active ingredient dosage, although collected, has not been considered in the statistical analysis, since inclusion of this variable led to a loss of power in the results and caused confusion, especially with respect to combined drug treatment. The supposition is that the severity of those patients who did not receive drug treatment was milder, since this is the protocol recommended by clinical practice guidelines [32,50]. However, this relationship cannot be established since this variable is not included in the computerized clinical history. Further studies are needed to examine the relationship between seriousness and the duration of the pharmacological treatment, since both variables may shed light on the phenomenon of recurrence of depression [32].

Other aspects to be considered as study limitations include the diagnosis of depression made by the family physician, since as previously mentioned, there is an under-diagnosis in primary care [43,44]; and the existence of comorbidities. In this study, only serious psychiatric comorbidities have been considered as exclusion criteria. As for comorbidity, a study by Gili et al [12] that compared the comorbidity of affective disorders and medical diseases in primary care patients with either a first or recurrent depressive episode, revealed that all medical conditions were more prevalent in the recurrent patients group than in the first-episode group (aOR = 2.61, CI = 2.31–2.93), after adjusting for gender, age, education, socioeconomic status and body mass index. In this study, the suitability of drug treatment based on patient comorbidities has not been examined, given that the objective is to describe depressive episodes in a primary care patient cohort and the administered pharmacologic treatment, from a naturalistic approach. Due to the universal nature of the healthcare system and the absence of other primary healthcare providers, it is known that subjects did not receive other pharmacological or psychological treatment from the healthcare system, but it is not known if they may have received some psychological therapy from outside of the national healthcare system. This may be considered a potential study limitation, since cognitive behavioral interventions, which may be carried out during the acute phase, appear to have a lasting protective effect on patients, preventing relapse and perhaps, recurrence upon treatment termination [3]. Finally, the sample has consisted mainly of female patients, as expected in Mediterranean countries in which women are almost three times more likely to suffer from MDD than men [57]. This fact, together with the aging population may further limit the generalization of our data. However, no clear influence of gender on antidepressant response has been reported in literature, at least for outpatients [58] and an aging population is a common demographic phenomenon in many developed countries.

## Conclusion

Recurrence of depressive episodes is frequent in primary care patients, affecting more than 40% of the population, being more common in women. Monotherapy and specifically SSRIs were the most frequently prescribed treatment option for all depressive episodes. As for combined pharmacologic treatment, the most frequently prescribed drug combinations were SSRIs and benzodiazepines.
